# Policy decisions matter: Cessation of logging benefits 34 threatened species in Victoria, Australia

**DOI:** 10.1371/journal.pone.0319531

**Published:** 2025-03-12

**Authors:** Kita R. Ashman, Michelle Ward, Chris R. Dickman, Dan Harley, Leonie Valentine, John Woinarski, Jess R. Marsh, Chris J. Jolly, Don A. Driscoll, Elle Bowd, Darcy J. Watchorn, Nick Clemann, David B. Lindenmayer

**Affiliations:** 1 Regenerative Country, WWF-Australia, Melbourne, Victoria, Australia; 2 Fenner School of Environment & Society, The Australian National University, Canberra, ACT, Australia; 3 School of Earth and Environmental Sciences, The University of Queensland, Brisbane, Queensland, Australia; 4 School of Environment and Science, Griffith University, Nathan, Queensland, Australia; 5 School of Life and Environmental Sciences, The University of Sydney, Camperdown, New South Wales, Australia; 6 Wildlife Conservation & Science, Zoos Victoria, Parkville, Victoria, Australia; 7 University of Western Australia, School of Biological Sciences, The University of Western Australia, Crawley, Western Australia, Australia; 8 Research Institute for the Environment and Livelihoods, Charles Darwin University, Darwin, Northern Territory, Australia; 9 Harry Butler Institute, Murdoch University, Murdoch, Western Australia, Australia; 10 School of Natural Sciences, Macquarie University, Sydney, New South Wales, Australia; 11 School of Life and Environmental Sciences, Deakin University, Burwood, Victoria, Australia; University of Ferrara, ITALY

## Abstract

In January 2024, the Australian state of Victoria committed to ending native forest logging six years ahead of schedule, a decision that has been advocated for by scientists and conservationists for decades. However, the direct benefits for threatened species from this policy change has not been quantified. This study assesses the spatial overlap between areas approved for logging and the habitats of nationally listed threatened species, to estimate the potential impacts of continued logging and the likely benefits of its cessation. We found that 99% of the areas approved for logging overlapped with the habitats of nationally threatened species. On average, each logging cutblock contained habitat for eight listed species. Areas approved for logging had considerable overlap with the habitat of several threatened species, particularly the Baw Baw frog (*Philoria frosti,* Critically Endangered, 6.2% of habitat approved for logging), Leadbeater’s possum (*Gymnobelideus leadbeateri*, Critically Endangered, 6.1%), barred galaxias (*Galaxias fuscus*, Endangered, 5.6%), Tall astelia (*Astelia australiana*, Vulnerable, 5.4%), and Colquhoun grevillea (*Grevillea celata*, Vulnerable, 5%). Notably, these five species are found only in Victoria, thus these values represent the proportion of their entire mapped habitat slated for logging over a short time period. Our findings underscore the need for urgent, nationwide forest protection policies, alongside restoration efforts, to support species recovery and meet global climate and biodiversity commitments.

## Introduction

Despite their importance for biodiversity and climate mitigation, forests are among the most disturbed terrestrial ecosystems globally [1]. At least a third of Earth’s forests have been destroyed, and a much higher proportion of remaining forest is degraded [2], with key drivers being agriculture and logging [3]. The impacts of logging on forest systems are substantial. Logging: i. alters fire regimes [4], and contributes to megafires [5,6], ii. facilitates degradation of water quality [7], iii. reduces carbon capture and sequestration potential of forests and soils [8,9], iv. removes key habitat elements such as hollow-bearing trees and reduces habitat suitability for many species [10], v. increases the prevalence of disease [11] and, vi. promotes populations of invasive species [12,13]. Although these impacts are well-understood, logging has continued throughout some of the world’s most important areas for conservation, and commitments made by governments and corporations to curb forest destruction have generally achieved very little to date [14]. At the COP26 summit in 2021, 137 world leaders committed to ending and reversing deforestation by 2030. However, very few countries have taken meaningful action to achieve this goal, such as committing to end native forest logging.

Australia’s native forest cover has declined significantly since European invasion, and logging has occurred extensively in eucalypt forests in the Australian states of Western Australia, New South Wales, Queensland, Tasmania, and Victoria [15]. In May 2023, the Victorian government announced a commitment to end native forest logging across the state by 1 January 2024, six years earlier than a previous commitment to stop logging by 2030. This announcement acknowledged that ending logging serves both economic interests and environmental commitments. For example, Victoria’s Parliamentary Budget Office estimated that an immediate cessation of logging would save the Victorian budget $192 million by 2030 [16]. In addition to the economic merits, stopping logging is likely to have considerable benefits to communities; forests that remain intact represent important carbon sinks [8,9], generate clean drinking water [7], improve air quality [17], are typically less fire prone when compared to younger, logged forests [4], and can provide for recreation and ecotourism [18]. While there are numerous benefits arising from the cessation of logging, and the policy decision taken by the Victorian Government is significant, it should be noted that, to date, the commitment is not enshrined in current legislation and therefore could be subject to reversal should there be a change in government.

Despite scientists and conservationists seeking this policy decision for decades [19], the benefits arising for threatened species as a result of the cessation of logging (i.e., habitat loss avoided) has not been quantified. Impact evaluations are a key step in determining whether such policy step changes are effective and can provide important opportunities for improving their application. Here, using Victoria as a case study, we assess the extent and location of previously approved logging in the State’s most recent Timber Release Plan. The plan was in effect from 2021–2026 and outlines the specific areas, methods, and timing of native forest logging that was approved and scheduled to be logged. We then examine spatial overlap between areas previously approved for logging and the mapped habitats of nationally listed threatened species to estimate the habitat loss that would have occurred if logging had continued, and the benefits that have accrued to threatened species as a consequence of the policy decision to cease logging. We note that these estimates of potential impact extend from impacts arising from many decades of previous logging in these forests: much habitat for many of the threatened species considered here has already been affected.

The key questions we investigate are

– What is the extent of spatial overlap between mapped habitat for nationally listed threatened species and areas that were previously approved for logging across the state of Victoria?
– Which threatened species distributions had the greatest spatial overlap with areas that were approved for logging, and thereby which species will benefit the most from this policy decision?

Our evaluation does not consider additional habitat destruction that would have occurred in any next round of logging between 2026 and 2030 (the previously defined endpoint for logging in these forests) or take account of the legacy effects from the habitat previously impacted by logging prior to 2021. We discuss the implications of our spatial analysis and present the case for policy and decision makers to prevent further destruction of native forests in other Australian States and Territories, and globally. We also emphasise the importance of enshrining such policy decisions in legislation to reduce the likelihood of future policy reversal following changes of government.

## Methods

### Study region

Our study encompassed the State of Victoria, an extensively deforested state in south-eastern Australia, where more than half (~15.6 million hectares) of all native forest has been destroyed in the past 200 years since European invasion [20]. Victoria supports approximately 6.9 million hectares of remaining forest on public land. These areas comprise ~ 3.7 million hectares of protected areas including parks and conservation reserves, and ~ 3.2 million hectares of what is considered ‘multiple use areas’, which includes areas that were approved for logging [21]. This ~ 3.2 million hectares of remaining forest cover can be categorised into five broad types based on tree species composition: i. montane ash forest, ii. low elevation mixed species, iii. high elevation mixed species, iv. box-ironbark, and v. river red gum forests [22]. Logging has typically been concentrated in montane ash forests, which are dominated by mountain ash (*Eucalyptus regnans*) and alpine ash (*Eucalyptus delegatensis*), as well as across mixed species forests both at low and high elevations [22].

### Species distribution data

Victoria supports 267 nationally listed threatened species, comprising 106 vertebrate species (24 mammals, 47 birds, 12 reptiles, 10 amphibians, and 13 fishes), seven invertebrate species, and 154 plant species [23]. In addition, Victoria has a high degree of endemism with an abundance of unique flora, fauna, and fungi. Notably, the number of listed invertebrate and fungi species will be a vast underestimate of those that are threatened, reflecting significant knowledge gaps surrounding these groups. For the purposes of this paper, we considered a species to be ‘threatened’ if it had been assigned the status of Vulnerable, Endangered, or Critically Endangered under the Environment Protection and Biodiversity Conservation (EPBC) Act 1999 - Australia’s national environmental legislation.

We obtained spatial data layers on the predicted distributions of threatened flora and fauna habitats from the Australian Government Species of National Environmental Significance (SNES) spatial database (accessed 15/01/2024) [24]). The layers are publicly available grids (1 km^2^ – 10 km^2^ resolution) of Maxent (a program for modelling species distributions from presence-only species records) modelled areas of occurrence of species listed under the EPBC Act. Data are coded as ‘Species habitat likely to occur’, which includes known records, and ‘Species habitat may occur’ which includes both known and unverified records. We extracted only ‘likely to occur habitats’ of all nationally listed threatened species from the SNES spatial grids. All spatial layers were projected to the coordinate system VICGRID2020. While these data are relatively coarse compared to single species distribution models, the SNES data are the spatial data used to assess the potential impact on threatened species from referred projects (e.g., development projects, logging, mineral extraction etc.) under the EPBC Act. Therefore, we considered this to be the most relevant spatial dataset with respect to our current environmental protection procedures.

### Forest logging data

In Victoria, a Timber Release Plan is a spatial dataset that is periodically updated and delineates areas where commercial logging on public land has been approved and may occur during a specified timeframe (in this case, 2021–2026, data accessed 15/01/2024) [25]). We filtered the Timber Release Plan data to include only cutblocks (defined as an area of forest where logging is planned to occur) with the status ‘current’ for our analysis. We omitted six records with the status ‘log store’, along with 729 records with the status ‘current regen’. The total number of cutblocks used in our analyses was 1,828, with each varying in area from 0.6 to 251.8 ha (median 38.4 ha) and covering a total combined area of 70,285 hectares, all of which was approved for future logging between 2021–2026.

### Overlap of approved logging and habitat for threatened species

All analyses were completed in ArcGIS, ArcMap version 10.8 [26]. Spatial analyses in ArcGIS are widely regarded for their scientific rigor and validity, owing to the platform’s robust suite of tools designed to handle a variety of spatial data types and complex analytical tasks. The software adheres to established geospatial methodologies and supports rigorous data processing, visualisation, and modelling. Furthermore, the system allows for the integration of statistical tools and spatial autocorrelation methods, enhancing the reliability and reproducibility of results, reinforcing the validity of spatial analyses in informing conservation decisions.

We used the Calculate Geometry tool (accessible via the attribute table or the geoprocessing pane) to calculate the area of habitat for all species in the SNES shapefile to create Australia-wide habitat range estimates. Following this national analysis, we focused on the state of Victoria. We applied the clip tool in ArcMap to extract only the relevant SNES data corresponding to the geographical boundaries of Victoria. Once the dataset was filtered, we recalculated the habitat area for each species, using the same spatial analysis procedures as in the initial calculation, to derive accurate estimates of habitat cover specifically within Victoria. With both the national and state-level habitat area values in hand, using the Tabulate Intersection tool, we computed the proportion of habitat present in Victoria for each threatened species. This step was crucial for our analysis, as it allowed us to understand the relative habitat availability in Victoria compared to the national level. We established a threshold by excluding species that had less than 1% of their total habitat located within Victoria. As a result of this threshold process, we eliminated 68 species from further analyses, while retaining 319 species for a more focused examination.

To eliminate potential errors stemming from duplicate boundaries of cutblocks, we executed a dissolve analysis using the Dissolve Boundaries tool on the filtered Timber Release Plan data (areas designated with a “current” status only). This process merged overlapping boundaries that shared attributes, thereby simplifying the dataset and removing the possibility for duplication.

Following the dissolution of the overlapping boundaries, we proceeded to further refine our analysis by clipping the filtered species data—specifically, those species with more than 1% of their habitat located in Victoria—against the newly dissolved Timber Release Plan data. This clipping operation allowed us to isolate the habitat areas of these species that directly intersect with the Timber Release Plan, enabling us to calculate the extent of mapped habitat for each species in relation to the planned logging activities. To assess the conservation implications of timber management on biodiversity, we then calculated the proportion of each species’ habitat that overlaps with the Timber Release Plan. Based on this analysis, we established a threshold for further investigation, excluding any species that exhibited less than 1% habitat overlap with the Timber Release Plan from ongoing analyses. As a result, we removed 105 species, retaining a focus on 34 species that showed notable habitat overlap.

Lastly, to gain insights into the number of species with mapped habitats that intersect with individual cutblocks within the Timber Release Plan, we performed a spatial join (using the Spatial Join tool in the geoprocessing toolbox). This technique combined the attribute data from the SNES species dataset with the original (undissolved but filtered to only “current” status) Timber Release Plan data, allowing us to accurately identify and tally the species present in each cutblock boundary. This step was crucial for understanding the potential impacts of logging activities at the cutblock level.

## Results

**Fig 1:**
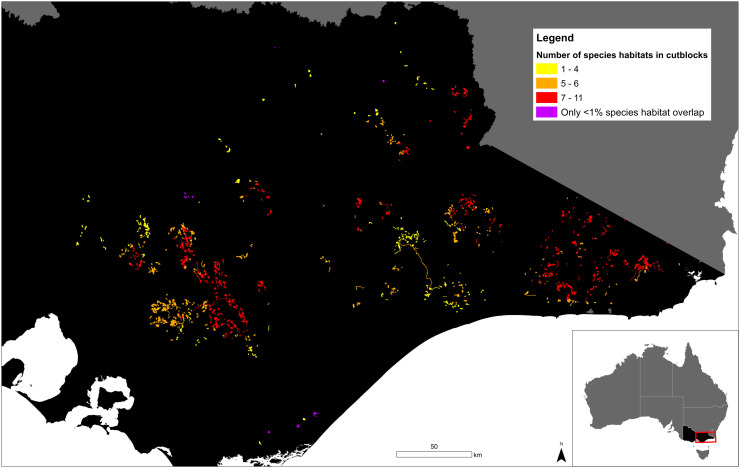
Distribution of former cutblocks within Victoria’s previously approved Timber Release Plan with the number of listed threatened species mapped habitats overlapping shown in yellow (1–4 species habitats overlapping), orange (5–6 species habitats overlapping), and red (7–11 species habitats overlapping). Cutblocks with overlap of only species < 1% mapped habitats in Victoria are shown in purple.

All cutblocks in the Timber Release Plan overlapped with the mapped habitat of at least one nationally listed threatened species (Table 1 in S1 File). After filtering the data to remove species with < 1% habitat in Victoria and < 1% habitat overlapping the Timber Release Plan, 99% (1809/1828 cutblocks, or 69,355/70,285 hectares) of cutblocks still overlapped with the habitat of at least one nationally listed threatened species. Most cutblocks included habitat for multiple threatened species, with a mean of eight and a maximum of 11 listed threatened species (Figure 1). The highly restricted Baw Baw frog (*P. frosti*) had the greatest spatial overlap, with 6.2% of its range overlapping areas that had been approved for logging between 2021 and 2026 (Table 1). Other species which had high spatial overlap include Leadbeater’s possum (*G. leadbeateri;* 6.1%), barred galaxias (*G. fuscus*; 5.6%), Tall astelia (*A. australiana*; 5.4%), and Colquhoun grevillea (*G. celata*; 5%) (Table 1). Notably, these five species are only found in Victoria, meaning that these values represent the proportion of their entire mapped habitats potentially impacted by logging within the 5-year period.

**Table 1:** Listed threatened species with the top 10 highest spatial overlap with previously approved timber release plan in the state of Victoria. Full list of all species with spatial overlap provided in S1 File ‘% in TRP’ corresponds to the Victorian range of the listed species (which in some cases may be the entire range).

Common name	Scientific name	EPBC status	Taxon group	Ha nationally	Ha in Vic	% in Vic	Ha in TRP	% in TRP
Baw Baw Frog	*Philoria frosti*	Critically Endangered	frogs	78,151	78,151	100	4,855	6.2
Leadbeater’s Possum	*Gymnobelideus leadbeateri*	Critically Endangered	mammals	433,034	433,034	100	26,273	6.1
Barred Galaxias	*Galaxias fuscus*	Endangered	fishes	86,520	86,520	100	4,874	5.6
Tall Astelia	*Astelia australiana*	Vulnerable	flora	47,334	47,334	100	2,537	5.4
Colquhoun Grevillea, Nowa Nowa Grevillea	*Grevillea celata*	Vulnerable	flora	10,179	10,179	100	507	5.0
Coughran’s Crayfish, Arte Spiny Crayfish	*Euastacus sp. 1 coughrani (McCormack & Fetzner)*	Endangered	other-animals	53,635	53,635	100	2,087	3.9
West Gippsland Galaxias	*Galaxias longifundus*	Critically Endangered	fishes	19,486	19,486	100	634	3.3
No recorded common name	*Olearia rugosa subsp. distalilobata*	Endangered	flora	46,294	44,816	96.8	1,103	2.5
Leafless Tongue-orchid	*Cryptostylis hunteriana*	Vulnerable	flora	4,263,815	396,089	9.3	9,324	2.4
Alpine Leafy Liverwort	*Pseudocephalozia paludicola*	Vulnerable	flora	46,0952	9,965	2.2	222	2.2

## Discussion

Spatial conflict between resource extraction and high conservation value areas is a pressing global issue, as competing demands for land and natural resources threaten biodiversity and ecological integrity [27,28]. Increasing demand for natural resources often leads to the encroachment of extractive activities into high conservation value areas, such as critical habitats, climate refugia, and biodiversity hotspots [29,30]. This conflict poses significant challenges to meeting sustainable development goals, as it often results in habitat destruction, fragmentation, and the degradation of ecosystems, jeopardising the persistence of numerous species [27,29]. Striking a balance between economic interests and environmental protection and preservation remains a key challenge in addressing the spatial conflict and ensuring the long-term health of our planet. However, in Australian native forestry, where the costs of logging exceed the returns [31], Victoria’s announcement to end native forest logging signifies a win-win with improvements economically and environmentally. While the commitment has marked a pivotal moment in the State’s environmental stewardship, the announcement is yet to be followed up with changes to legislation, or the declaration of new protected areas. As a result, the commitment could be subject to reversal if there is a change in government [31].

We found an almost complete (>99%) overlap between the areas that were approved for logging until 2026 in the Timber Release Plan and the mapped habitats of nationally listed threatened species across the Australian State of Victoria. On average, each cutblock encompassed the mapped habitat of eight listed threatened species. The habitat of several threatened species had considerable overlap with areas approved for logging in the 2021 – 2026 timber release plan, most notably the Baw Baw frog, Leadbeater’s possum, barred galaxias, tall astelia, and Colquhoun grevillea. This highlights the habitat retention benefits arising for these threatened species as a result of the policy decision to cease logging. However, we note that the habitat protected for threatened species relates solely to areas originally proposed for logging over the period 2021 to 2026 and does not consider additional habitat destruction that would have occurred in any next round of logging between 2026 and 2030. Furthermore, our evaluation does not take account of the legacy effects from the habitat previously impacted by logging prior to 2021. The benefits for species and habitats arising from cessation of logging will vary based on factors like life history, habitat requirements, and critical habitat areas [32]. However, directly mitigated impacts are likely to include the retention of key habitat structures such as large, old hollow bearing trees, upon which numerous species depend [33,34], especially hollow-dependent species like Leadbeater’s possum and the southern greater glider, both of which had a high degree of spatial overlap with areas previously logged and those proposed to be logged between 2021-2026 (Table 1).

### The urgent need for cessation of logging

The cessation of industrial scale native forest logging in Victoria and Western Australia, has broader implications for conservation policy both in Australia and globally. In Australia, this precedent could offer opportunities for other states to follow suit, such as New South Wales, and Tasmania, where logging is still having a significant impact on nationally listed threatened species. These listed species are already on a trajectory of decline, often at an alarmingly rapid rate [35]. For example, under the EPBC Act, Critically Endangered species have met criteria that broadly correspond to having ≥ 50% chance of extinction in the wild within the next decade (or 3 generations, whichever is longer), species listed as Endangered as having ≥ 20% chance of extinction in 20 years (or 5 generations), and Vulnerable species as having ≥ 10% chance of extinction in the next 100 years [36]. Despite the urgency to halt the decline of species listed in these categories, very little has been done in New South Wales and Tasmania to limit extractive and destructive management practices such as logging that operate at a landscape-scale, causing significant habitat destruction [37–39]. As a case in point, the southern greater glider was listed as vulnerable in 2016, at which time logging was cited as a key threat to the persistence of populations [40]. In the years following this listing, anthropogenic climate induced fires impacted at least a third of the species’ range and its status was uplisted to Endangered in 2022 [39,40]. However, since that time, logging has continued across much of the southern greater glider’s range, including within key unburnt habitat [41]. Removing logging as a threat nationally will be essential for reducing the extinction risk for this and many other forest-dependent threatened species.

Globally, the need for policies that remove native forest logging as a threat is becoming increasingly urgent as more regions face mounting pressure to balance environmental conservation with economic development and extractive industries [19]. In the Himalaya region within Sharda (Neelum valley), indiscriminate deforestation for fuel, medicinal resources, and development, have led to the reduction of forest area, threatening the viability of important indigenous flora [19]. Direct conservation measures including the removal of practices contributing to deforestation, including logging, are urgently required [19,42]. Similar interventions ceasing logging in temperate forests in the Pacific Northwest and the Amazon, have had mixed results depending on the strength of local governance and enforcement mechanisms [43]. Ending native forest logging across countries could yield long-term benefits like improved biodiversity, enhanced ecosystem services, and greater climate resilience [43,44]. However, challenges include industry resistance, varying economic dependence on forestry, and the need for strong enforcement [19,43]. Successful implementation of forest preservation policies will require alternative livelihoods, effective land-use planning, and investment in forest restoration and sustainable management practices [19,43].

### Ongoing issues and loopholes with logging in Victoria

Although the Victorian government has indicated that it planned to end native forest logging at the start of 2024, the specific details of the policy may allow some logging to continue. There are plans for extensive post-disturbance salvage logging following windstorms (and also likely after wildfires) in Victorian forests [45]. The negative ecological impacts of salvage logging are well documented [46], such that salvage logging is currently being considered for listing as a threatening process under State legislation (i.e., the Victorian *Flora & Fauna Guarantee Act* 1988). In addition, within the remaining fragmented forests, there is uncertainty on how ‘forest gardening’, a term used to describe a collective of management practices aimed at restoring First Nations Country, that can include conventional logging and thinning, will impact threatened species.

While uncertainty remains regarding what will happen to Victoria’s state forests following the end of native logging, many areas could be designated as new protected areas. This includes forests that have been previously logged and degraded where high-quality restoration programs developed in partnership with First Nations custodians could be implemented to achieve financially, socially, and environmentally sustainable outcomes. These restoration programs could provide working models for the Victorian government, First Nations groups and other stakeholders to replicate the methods at scale and, in turn, build resilience across thousands of hectares of threatened species habitats.

### Required changes to forest policy nationally

There is an urgent need to address conflicting legislation at different levels of government in Australia. To date, logging of native forests throughout Tasmania and New South Wales, and up until recently in Victoria and Western Australia, has been exempt from threatened species impact assessments under the Environment Protection and Biodiversity Conservation Act by virtue of Regional Forest Agreements (RFAs) [47]. These agreements between state and federal governments, fail to adequately consider the conservation needs of numerous threatened species, such as those that have been listed as threatened after agreements were signed in early 2000. The RFA’s also fail to take into account changed circumstances for numerous species following major bushfires resulting in significant habitat loss, as occurred in 2019/20 [48]. Australia has recently committed to national and international targets that include agreements to halt species extinctions (e.g., Threatened Species Action plan; Convention of Biological Diversity), prevent further forest degradation (Glasgow Climate Pact), and reverse biodiversity decline (e.g., Natures Pledge). Despite these agreements, there has been no commitment by the Australian, New South Wales or Tasmanian governments to end native forest logging, despite this providing one of the main mechanisms for meeting these obligations. In Victoria, the current policy to cease logging has yet to be enshrined in legislation and therefore could be subject to reversal. To ensure Australia meets its commitments on biodiversity, climate mitigation and forest protection, major reform is required to resolve these legislative inconsistencies. Given the high number of overlapping mapped threatened species habitats in East Gippsland, the Central Highlands and north-eastern Victoria (Fig 1), these areas should be targeted as a priority for immediate formal protection and active restoration. The protection and restoration of such habitats in the first instance will be key for supporting viable populations of species in an increasingly unstable climate.

### Technical caveats and considerations

This study examines only one specific impact of native forest logging on biodiversity, the direct overlap of mapped threatened species habitats with areas formerly approved for logging. As such, the magnitude of impacts described, which are significant for several threatened species, are nonetheless an underestimate of the full ecological impacts arising from logging and fail to account for downstream or indirect impacts that logging has on species and habitats and disruption of ecological processes. In addition, the estimates of habitat destruction generated cover the 5-year period 2021-2026 and do not take account of the extensive logging impacts on many species that occurred prior to 2021. Moreover, hyper diverse taxa, and functionally important taxa, such as invertebrates and fungi, often have substantial data deficiencies, meaning numerous imperilled and declining species are not listed under formal environmental legislation [49–51]. Consequently, the full ecological benefits from ceasing logging are not represented in our analyses but are considerable.

An important caveat concerning the 94,855 hectares of forest formerly approved for logging is that the entire area was unlikely to be harvested within the approved timeframe (2021–2026). However, the full area would be available for logging over subsequent years, so this doesn’t necessarily alter the extent of impact on threatened species, just the time over which those impacts occur. Victoria’s state-owned logging agency claimed to harvest an average of 3,000 ha annually [52]. However, state government annual reporting data suggests that the area harvested each year is often up to three times higher than this average [39]. Irrespective of the actual scale of logging operations which were occurring across Victoria, logging throughout the State was often concentrated in some of the most important areas for biodiversity conservation, such as unburnt forest providing critical refugia [53]. As a result, undisturbed, high-quality forest patches have become smaller and more dispersed at the landscape level [22,53]. Accordingly, the impacts, and associated benefits of ending logging for certain species, particularly rare, disturbance-sensitive, specialist and range-restricted species, may be higher than that indicated by the proportion of overlap that was available for logging [37,54].

## Conclusions

This study demonstrates that ending native forest logging in Victoria will directly benefit at least 34 nationally listed threatened species by removing the ongoing threat of logging within much of their habitats. The cessation of logging also offers broader ecological benefits, including improved carbon storage [8,9], water quality [7], and air quality [17], along with reduced fire risks and increased opportunities for ecotourism [20,23]. However, to secure these benefits, the policy must be enshrined in legislation to prevent future reversals, particularly in the face of political changes [55].

Our findings highlight the urgent need for national policy reform to address the gaps in environmental protections that currently allow continued habitat destruction [37,39]. Effective protections should account for cryptic and data-deficient species [56], as well as the cumulative effects of logging, fire, and climate change [54,56]. To meet global biodiversity and climate goals, policymakers must prioritise the formal protection of native forests and invest in scalable, sustainable restoration programs, developed in partnership with First Nations custodians [57]. These findings are not only relevant to Victoria but could serve as a model for similar interventions in other regions, emphasising the potential for replication in broader global efforts to halt deforestation, biodiversity loss and combat climate change.

## Supporting information

S1 Table Full list of all species considered in analysis with >1% range within Victoria and >1% spatial overlap with current timber release plan. S2 Table. Number of listed threatened species mapped habitats overlapping with cutblocks included in current timber release plan.
