# Supplementary material for: Policy decisions matter: Cessation of logging benefits 34 threatened species in Victoria, Australia
Source: PLoS One. 2025 Mar 12;20(3):e0319531. doi: 10.1371/journal.pone.0319531 (PMC11902118; doi:10.1371/journal.pone.0319531)
Supplement: S1 Table — S2 Table. Number of listed threatened species mapped habitats overlapping with cutblocks included in current timber release plan. [file pone.0319531.s001.docx]

**Supplementary material**

**S1 Table. Full list of all species considered in analysis with >1% range within Victoria and >1% spatial overlap with current timber release plan.**

| **Common name** | **Scientific name** | **EPBC status** | **Taxon group** | **Ha nationally** | **Ha in Vic** | **% range in Vic** | **Ha in TRP** | **% range in TRP** |
| --- | --- | --- | --- | --- | --- | --- | --- | --- |
| Baw Baw Frog | *Philoria frosti* | Critically Endangered | Frog | 78,151 | 78,151 | 100 | 4,855 | 6.2 |
| Leadbeater's Possum | *Gymnobelideus leadbeateri* | Critically Endangered | Mammal | 433,034 | 433,034 | 100 | 26,273 | 6.1 |
| Barred Galaxias | *Galaxias fuscus* | Endangered | Fish | 86,520 | 86,520 | 100 | 4,874 | 5.6 |
| Tall Astelia | *Astelia australiana* | Vulnerable | Flora | 47,334 | 47,334 | 100 | 2,537 | 5.4 |
| Colquhoun Grevillea, Nowa Nowa Grevillea | *Grevillea celata* | Vulnerable | Flora | 10,179 | 10,179 | 100 | 507 | 5.0 |
| Coughran's Crayfish, Arte Spiny Crayfish | *Euastacus sp. 1 coughrani* | Endangered | Invertebrate | 53,635 | 5,3635 | 100 | 2,087 | 3.9 |
| West Gippsland Galaxias | *Galaxias longifundus* | Critically Endangered | Fish | 19,486 | 19,486 | 100 | 634 | 3.3 |
| No recorded common name | *Olearia rugosa subsp. distalilobata* | Endangered | Flora | 46,294 | 44,816 | 96.8 | 1,103 | 2.5 |
| Leafless Tongue-orchid | *Cryptostylis hunteriana* | Vulnerable | Flora | 4,263,815 | 396,089 | 9.3 | 9,324 | 2.4 |
| Alpine Leafy Liverwort | *Pseudocephalozia paludicola* | Vulnerable | Flora | 460,952 | 9,965 | 2.2 | 222 | 2.2 |
| Orbost Spiny Crayfish | *Euastacus diversus* | Endangered | Invertebrate | 154,540 | 154,540 | 100 | 3,307 | 2.1 |
| Long-footed Potoroo | *Potorous longipes* | Endangered | Mammal | 701,889 | 668,541 | 95.2 | 13,902 | 2.1 |
| Dwarf Violet | *Viola improcera* | Endangered | Flora | 40,581 | 9,363 | 23.1 | 193 | 2.1 |
| Broad-toothed Rat (mainland), Tooarrana | *Mastacomys fuscus mordicus* | Endangered | Mammal | 2,236,507 | 1,410,953 | 63.1 | 28,837 | 2.0 |
| Roundsnout Galaxias | *Galaxias terenasus* | Endangered | Fish | 453,783 | 156,589 | 34.5 | 3,135 | 2.0 |
| Watson's Tree Frog | *Litoria watsoni* | Endangered | Frog | 1,672,415 | 665,900 | 39.8 | 13,317 | 2.0 |
| Greater Glider (southern and central) | *Petauroides volans* | Endangered | Mammal | 40,742,874 | 2,899,028 | 7.1 | 57,570 | 2.0 |
| Lemon-scented Zieria | *Zieria citriodora* | Vulnerable | Flora | 33,111 | 12,648 | 38.2 | 244 | 1.9 |
| Mountain Skink | *Liopholis montana* | Endangered | Reptile | 1,957,764 | 1,229,099 | 62.8 | 23,207 | 1.9 |
| Black-faced Monarch | *Monarcha melanopsis* |  | Bird | 23,961,683 | 1,671,806 | 7 | 31,492 | 1.9 |
| Bidhawal/Bidawal Crayfish, East Gippsland Spiny Crayfish | *Euastacus bidawalus* | Endangered | Invertebrate | 412,280 | 315,074 | 76.4 | 5,867 | 1.9 |
| Smoky Mouse, Konoom | *Pseudomys fumeus* | Endangered | Mammal | 2,812,185 | 2,048,319 | 72.8 | 37,378 | 1.8 |
| Pilotbird | *Pycnoptilus floccosus* | Vulnerable | Bird | 7,995,752 | 3,426,666 | 42.9 | 62,359 | 1.8 |
| East Gippsland Galaxias | *Galaxias aequipinnis* | Critically Endangered | Fish | 39,247 | 39,247 | 100 | 677 | 1.7 |
| Alpine Bog Skink, | *Pseudemoia cryodroma* | Endangered | Reptile | 263,140 | 220,641 | 83.8 | 3,728 | 1.7 |
| Concave Pomaderris | *Pomaderris subplicata* | Vulnerable | Flora | 9,129 | 9,129 | 100 | 139 | 1.5 |
| Spotted Tree Frog | *Litoria spenceri* | Critically Endangered | Frog | 1,542,148 | 1,390,999 | 90.2 | 17,751 | 1.3 |
| Austral Toadflax, Toadflax | *Thesium australe* | Vulnerable | Flora | 18,052,735 | 1,282,063 | 7.1 | 16,310 | 1.3 |
| Aniseed Boronia, Galbraith's Boronia | *Boronia galbraithiae* | Vulnerable | Flora | 16,636 | 16,636 | 100 | 210 | 1.3 |
| Brumby Sallee | *Eucalyptus forresterae* | Endangered | Flora | 52,494 | 32,385 | 61.7 | 396 | 1.2 |
| Mignonette Leek-orchid, Cobungra Leek-orchid, Dense Leek-orchid | *Prasophyllum morganii* | Vulnerable | Flora | 197,545 | 197,545 | 100 | 2,404 | 1.2 |
| Blue-tongued Orchid, Kiandra Greenhood | *Pterostylis oreophila* | Critically Endangered | Flora | 1,812,628 | 729,366 | 40.2 | 8,602 | 1.2 |
| Southern Giant Burrowing Frog | *Heleioporus australiacus flavopunctatus* | Vulnerable | Frog | 4,574,477 | 967,869 | 21.2 | 10,993 | 1.1 |
| Alpine Tree Frog, | *Litoria verreauxii alpina* | Vulnerable | Frog | 844,111 | 365,285 | 43.3 | 3,752 | 1 |

**S2 Table. Number of listed threatened species mapped habitats overlapping with cutblocks included in current timber release plan.**

| **Number of listed species habitats overlapping** | **Number of cutblocks** | **% of cutblocks** |
| --- | --- | --- |
| 1 | 26 | 1.4 |
| 2 | 65 | 3.6 |
| 3 | 87 | 4.8 |
| 4 | 154 | 8.5 |
| 5 | 291 | 16.1 |
| 6 | 332 | 18.4 |
| 7 | 320 | 17.7 |
| 8 | 354 | 19.6 |
| 9 | 139 | 7.7 |
| 10 | 40 | 2.2 |
| 11 | 1 | 0.1 |
